# Characterization of Tachyplesin Peptides and Their Cyclized Analogues to Improve Antimicrobial and Anticancer Properties

**DOI:** 10.3390/ijms20174184

**Published:** 2019-08-26

**Authors:** Felicitas Vernen, Peta J. Harvey, Susana A. Dias, Ana Salomé Veiga, Yen-Hua Huang, David J. Craik, Nicole Lawrence, Sónia Troeira Henriques

**Affiliations:** 1 Institute for Molecular Bioscience, The University of Queensland, Brisbane, Queensland 4072, Australia; 2 Instituto de Medicina Molecular João Lobo Antunes, Faculdade de Medicina, Universidade de Lisboa, 1649-028 Lisboa, Portugal; 3 School of Biomedical Sciences, Faculty of Health, Institute of Health & Biomedical Innovation, Queensland University of Technology, Translational Research Institute, Brisbane, Queensland 4102, Australia

**Keywords:** tachyplesin, host defense peptide, anticancer, antimicrobial, antibiofilm, peptide-membrane interaction, structure-activity, model membranes, nuclear magnetic resonance solution structure

## Abstract

Tachyplesin I, II and III are host defense peptides from horseshoe crab species with antimicrobial and anticancer activities. They have an amphipathic β-hairpin structure, are highly positively-charged and differ by only one or two amino acid residues. In this study, we compared the structure and activity of the three tachyplesin peptides alongside their backbone cyclized analogues. We assessed the peptide structures using nuclear magnetic resonance (NMR) spectroscopy, then compared the activity against bacteria (both in the planktonic and biofilm forms) and a panel of cancerous cells. The importance of peptide-lipid interactions was examined using surface plasmon resonance and fluorescence spectroscopy methodologies. Our studies showed that tachyplesin peptides and their cyclic analogues were most potent against Gram-negative bacteria and melanoma cell lines, and showed a preference for binding to negatively-charged lipid membranes. Backbone cyclization did not improve potency, but improved peptide stability in human serum and reduced toxicity toward human red blood cells. Peptide-lipid binding affinity, orientation within the membrane, and ability to disrupt lipid bilayers differed between the cyclized peptide and the parent counterpart. We show that tachyplesin peptides and cyclized analogues have similarly potent antimicrobial and anticancer properties, but that backbone cyclization improves their stability and therapeutic potential.

## 1. Introduction

The host defense peptides (HDPs) tachyplesin I, II and III (TI, TII and TIII) are active against a broad range of Gram-negative and Gram-positive bacteria and fungi [1,2,3,4] and possess anticancer properties [5,6,7,8,9,10,11,12,13,14,15]. Each analogue was isolated from a different species of horseshoe crab, but they share high sequence homology (Table 1). TI, TII and TIII possess 17 amino acid residues, two disulfide bonds, a C-terminal α-amidation, and their structure is organized in a β-hairpin [2,3,16]. Like other HDPs [17], TI, TII and TIII possess an amphipathic secondary structure (i.e., positively charged and hydrophobic amino acids segregate into distinct clusters), thought to be essential for their antimicrobial activity.

Cationic amphipathic HDPs selectively target the anionic surfaces of microbes, rather than the neutral surface of host cells, and kill them by a mechanism that involves binding to and insertion into cell membranes. The initial binding is mediated by electrostatic attractions between the positively-charged residues of HDPs and the anionic microbial surface [18], and is followed by the insertion of hydrophobic residues into lipid membranes in a process that involves van-der-Waal’s interactions with the phospholipids [18,19].

Similar to bacterial cells, the surface of cancer cells is negatively-charged due to the increased expression and exposure of phospholipids containing the anionic phosphatidylserine (PS) headgroup [20,21,22,23]. In contrast, cell membranes of healthy mammalian cells are asymmetric and phospholipids containing PS-headgroups are found exclusively in the inner leaflet. Variations in the overall cell surface charge [24] in phospholipids with exposed [25,26] membrane fluidity and curvature [18,27], have been shown to modulate the selective toxicity of HDPs towards cancer cells. These differences regulate the affinity of peptides for cell membranes, the effective peptide-to-lipid ratio and the ability for HDP to kill cancerous cells or pathogens rather than healthy cells [28,29,30].

Several studies have investigated the activity, structure and mechanism of the action of TI, but few have examined the activity of TII and TIII. Early studies suggested that TI kills bacterial cells by a mechanism involving inner membrane permeabilization and the rapid efflux of K^+^ [31,32,33]. Later, TI was shown to translocate across lipid bilayers, cause a phospholipid flip-flop and form toroidal pores [33,34]. In cancerous cells, TI was reported to induce cell disruption and late apoptosis/necrosis [12,35]. Paredes-Gamero et al. proposed that the cancer cell death mechanism was dependent on the peptide dose: at high concentrations, the peptide-induced direct cell membrane disruption, and at lower concentrations, it activated intracellular cell death mechanisms [12].

Because of the expression of TI, TII and TIII in distinct species of horseshoe crab, we were specifically interested in comparing their structure, activity and mode-of-action. As these peptides have a potential application as anticancer and/or antimicrobial agents, we investigated whether backbone cyclization would increase the stability and maintain activity. Our studies show that TI, TII and TIII, and their cyclic analogues cTI, cTII and cTIII, have similar structures and activities against bacteria and cancerous cells. Backbone cyclization reduced the hemolytic activity and increased peptide stability while maintaining potent anticancer and antimicrobial activities. cTI and cTIII especially showed potential to be considered for the development of anticancer peptide-based drugs.

## 2. Results

### 2.1. Properties of Tachyplesin I–III and Their Cyclic Analogues

The amino acid sequences of TI, TII and TIII differ in positions 1 or 15, which can be a lysine or an arginine residue (Table 1). So far, TI is the most studied and the only analogue with reported structure calculations [36,37,38]. We were interested in comparing TI, TII and TIII and their backbone-cyclized analogues (cTI–III) to identify similarities and differences in their three-dimensional structure and stability; and to determine whether these characteristics affect the membrane interactions and biological activity of the peptides.

All peptides were synthesized using solid-phase peptide synthesis, oxidized and correctly folded, as suggested by the observed masses using electrospray ionization mass spectroscopy (ESI-MS; Supplementary Figure S1 and Table 1) and confirmed through clearly dispersed peaks in the amide region of their respective One-dimensional (1D) ^1^H NMR spectra [39]. The peptides were purified to >95%, as confirmed by analytical reverse-phase high-performance liquid chromatography (RP-HPLC; see chromatograms of pure peptides in Supplementary Figure S1a).

Despite minor differences, their overall hydrophobicity follows the trend cTI > cTII > cTIII > TI > TII > TIII (Table 1), as indicated by their retention time (RT) on analytical RP-HPLC (Supplementary Figure S1). The cyclic analogues appear to be overall more hydrophobic (less polar) than the parent peptides, which is consistent with the loss of the N-terminal charge and the C-terminal amidation resulting from cyclization.

### 2.2. Structure of Tachyplesins and Backbone-Cyclised Analogues

**Figure 1:**
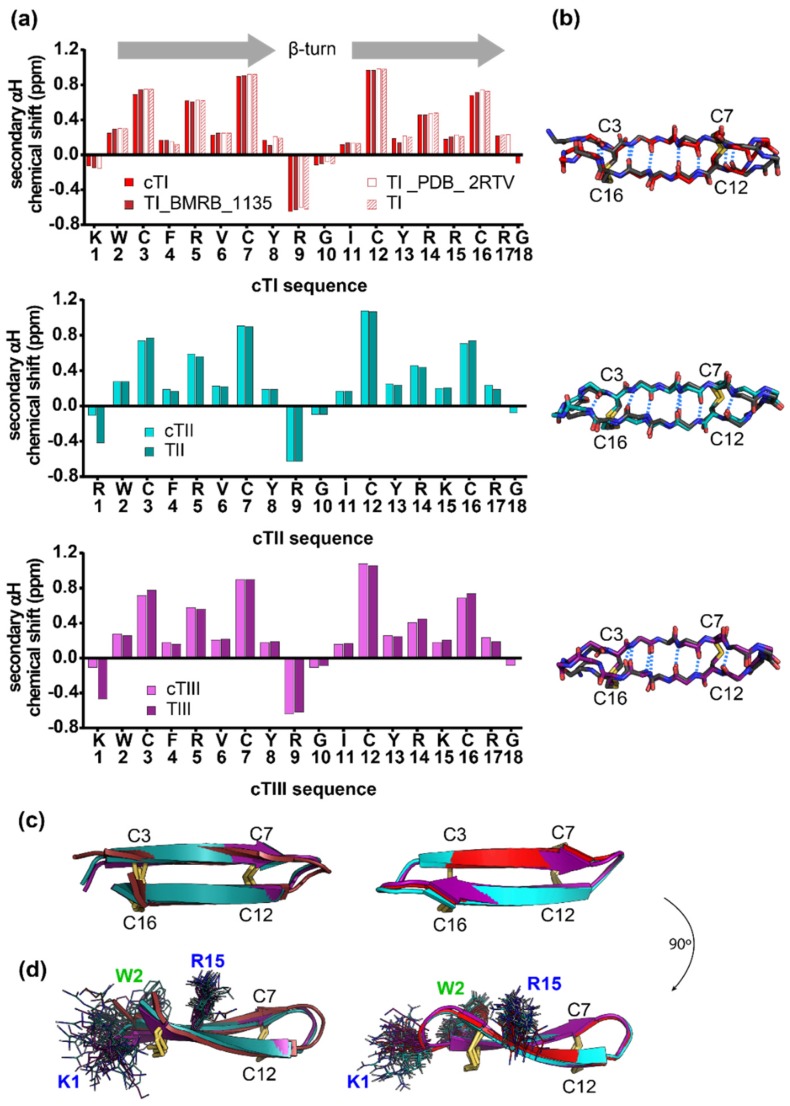
The NMR structures of TI and cTI (red), TII and cTII (cyan), TIII and cTIII (magenta). (**a**) Secondary αH chemical shift at 298 K determined from ^1^H NMR spectra. Shifts were calculated by subtraction of random coil ^1^H NMR shifts [41] from the experimental values. For tachyplesin I (TI), the shifts of the peptide synthesized in our lab were compared to shifts obtained from data banks: TI—PDB ID 2RTV [37], and TI—BMRB ID 1135 [38]. Positive shifts greater than 0.1 ppm suggest β-strands, indicated by grey arrows. (**b**) Overlay of the cyclic analogues (colored backbone) with their respective parent peptide (grey backbone). Hydrogen bonds between β-strands are indicated in blue. The structure of TI was obtained from the PDB (ID 1WO0). (**c**) Overlay of TI–TIII and cTI–cTIII. (**d**) Mobility of the residues (K or R) at position 1 and 15, which differ between the peptides, and of W2 of the native sequences and cyclic analogues, respectively.

The three-dimensional (3D) structures of TII and TIII, and of the backbone-cyclized analogues cTI–cTIII were determined with solution NMR spectroscopy. All backbone resonances were fully assigned apart from the N-terminal amides of the two linear peptides, and the R1/G18 amides of cTII, reflective of some degree of flexibility in these regions. The secondary αH chemical shifts of the native peptides and the cyclic analogues were highly similar, indicating a negligible change in the backbone structure and the β-strands (W2-Y8; I11-R17) (Figure 1a,b).

The 3D solution structures of each peptide were calculated from distance restraints, ranging from 154 for the linear peptides, to 172–284 for the cyclic analogues, along with dihedral angle restraints totaling 34 to 36. The final family of structures for each of the peptides has good structural and energy statistics, as indicated by an overall MolProbity score of less than 1.6, shown in Table S1. Analysis of the structures by PROMOTIF [40] defines antiparallel β-strands being formed by residues W2-Y8 and I11-R17 in all but one of the peptides. The exception is TIII which has slightly shorter strands formed between residues C3-Y8 and I11-C16. All disulfide bonds are defined by PROMOTIF as adopting the short right-hand hook configuration. Structures of the tachyplesin peptides and the cyclized analogues differed primarily in the flexibility of the N- and C-termini of the parent peptides (Figure 1b–d). The reduction of the amino acid side-chain flexibility in the terminal regions due to backbone cyclization is emphasized in Figure 1d with the side chain of the residues K/R1 and K/R15, which differ between TI/cTI, TII/cTII and TIII/cTIII; the side chain of W2 is also shown, as this residue is used to monitor the peptide partitioning into lipid bilayers (see Section 2.5.2). No significant cation-π interactions between the R/K1 and W2 were noted for any of the tachyplesin peptides. Such an interaction might be revealed by NMR in the form of substantial deviations of the chemical shifts of arginine/lysine residues but none were observed (see Figure 1a). The lack of cation-π interactions is also supported by the NMR solution structures that reveal a high degree of flexibility in the termini of the linear peptides, and by the different orientations that these residues acquire in the cyclic peptides, as shown in Figure 1d.

The peptide structures were deposited with the Protein Data Bank (PDB) and the Biological Magnetic Resonance Data Bank (BMRB): TII—PDB ID: 6PI2, BMRB ID: 30617; TIII—PDB ID: 6PI3, BMRB ID: 30618; cTI—PDB ID: 6PIN; BMRB ID: 30619; cTII—PDB ID: 6PIO, BMRB ID: 30620; cTIII—PDB ID: 6PIP, BMRB ID: 30621.

### 2.3. Improved Stability and Reduced Hemolytic Activity of Cyclized Tachyplesin Peptides

**Figure 2:**
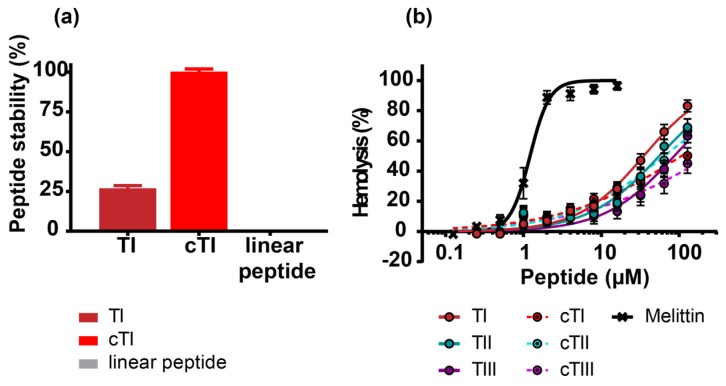
The peptide stability in human serum and activity against human red blood cells (RBCs). (**a**) Stability of TI and cyclic analogue cTI in 25% (*v*/*v*) human serum for 24 h. The samples were analyzed by analytical RP-HPLC and the percentage of peptide remaining was determined from the area under the peptide peak in the chromatogram in comparison to peak area at time zero. A linear 18 amino acid peptide (KGGGGSGQLIDSMANSFV) was included as the positive control. (**b**) Hemolytic activity of tachyplesins and their cyclic analogues were tested up to 128 µM against human RBCs (0.25% (*v*/*v*), 1 h incubation at 37 °C). Melittin, a highly hemolytic peptide, was included as the positive control.

Cyclization has been shown to increase stability [42,43] and reduce the hemolytic activity of some peptides [44,45]. Comparison of resistance to human proteases showed that the cTI analogue did not degrade after treatment for 24 h in 25% (*v*/*v*) human serum, whereas only 25% of TI remained in the solution (see analytical RP-HPLC chromatograms in Supplementary Figure S2). A peptide (linear and without disulfide bonds) used as a control was fully degraded under the same conditions (Figure 2a). TI has previously been shown to be completely stable for 2 h in mouse or human serum [46].

The percentage of hemolysis of human red blood cells (RBCs) followed the trend TI > TII > TIII > cTII > cTI > cTIII when compared at 128 µM, the highest concentration of peptide tested. TI was the most hemolytic of the native sequences. Backbone cyclization reduced the hemolytic activity of the cTI and cTIII compared to their parent counterparts, but led to no clear improvement for cTII. The C-amidated peptides TI–TIII lysed 66%, 56% and 41% of RBCs at 64 µM respectively (Figure 2b and Table 2). A similar trend but lower hemolytic activity had been reported for TI, TII, and TIII lacking C-terminal amidation at higher peptide concentrations [47], which is known to impact peptide activity [48,49].

The positive control melittin, a hemolytic peptide from honeybee venom, induces 100% hemolysis in human RBCs at concentrations above 2 µM. By comparison, at this concentration, all tachyplesin peptides have a low hemolytic activity of around 10%.

### 2.4. Biological Activity of Tachyplesin I–III and of Their Cyclic Analogues

#### 2.4.1. Activity against Bacteria

The Gram-negative *Escherichia coli* strains ATCC 25922 and DC2 CGSC 7139 and the Gram-positive *Staphylococcus aureus* strains ATCC 25923 and ATCC 6538 were used to determine the antimicrobial activity of the parent tachyplesin peptides and their cyclic analogues against bacteria with differences in the physical and chemical properties of their cell wall. *E. coli* ATCC 25922 (“smooth” LPS) and *S. aureus* ATCC 25923 are common control strains for antimicrobial susceptibility testing [50,51]. *E. coli* DC2 CGSC 7139 is hypersensitive to antibacterial agents and more permeable to dyes [52], while *S. aureus* ATCC 6538 is known to form biofilms [53]. Tachyplesin peptides and their cyclic analogues were tested against planktonic cultures of all strains and against *S. aureus* ATCC 6538 in the biofilm form for direct comparison of their antimicrobial activity.

Tachyplesin I–III were two-to-four times more potent than their cyclic analogues against all bacterial strains in their planktonic growth form (Table 3). Generally, the Gram-negative strains were more susceptible than the Gram-positive strains. Similar MICs have been reported previously for TI against *E. coli* ATCC 25922, [1] and TI and TII against *S. aureus* ATCC 25923 [2,3]. Tachyplesin peptides were most active against *E. coli* DC2 CGSC 7139 and *E. coli* ATCC 25922. The activity against these bacterial strains was reduced when the peptides were cyclized. The peptides were least active against *S. aureus* ATCC 6538 in the planktonic form, and no difference in activity was observed between tachyplesin peptides and cyclic analogues (Table 3).

**Figure 3:**
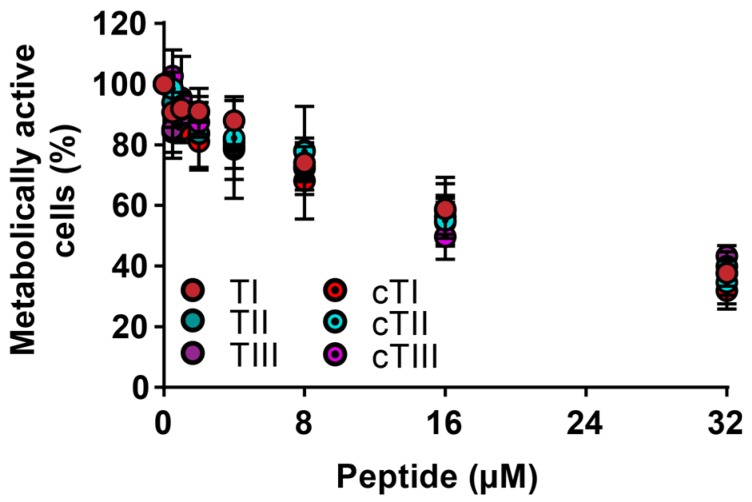
The activity of tachyplesin peptides and their cyclic analogues against an *S. aureus* ATCC 6538 biofilm. The response curve of biofilm treated with increasing the concentrations of peptide (*n* = 4, ± SEM).

To determine whether the tachyplesin peptides could act against bacterial biofilms, the cells metabolic activity was measured for biofilms formed by *S. aureus* ATCC 6538 (Figure 3 and Table 4). All peptides exhibited similar activity against the bacteria in the biofilm form, with a 50% loss of cell metabolic activity observed at ~20 µM. However, approximately 40% of the biofilm was metabolically active at 32 µM, the highest concentration tested, suggesting a reduced activity against biofilm compared to the planktonic *S. aureus* ATCC 6538 bacteria.

#### 2.4.2. Activity against Cancerous Cells

TI, TII, TIII, and their cyclic analogues were tested against three melanoma (MM96L, HT144 and WM164) and one cervical cancer (HeLa) cell line (Supplementary Tables S2 and S3) to determine whether the peptides exhibit different cytotoxic activities. The aneuploid immortal keratinocyte cell line HaCaT was included as a non-cancerous control. Cytotoxicity toward cancer cell lines was compared by determining peptide concentrations required to achieve 50% of cell death (CC50) from dose-response curves (Table 5).

The cytotoxic activities of the tachyplesin peptides and their cyclic analogues are dependent on the cell lines (Table 5). All peptides were most effective against the melanoma cell lines MM96L, HT144 and WM164 (cytotoxic activities ranged between CC50 0.8–2.7 µM). Compared to the control cell line HaCaT, the cytotoxic activities of the peptides against melanoma were significantly different (*p* < 0.05) with the exception of cTII against WM164. The cervical cancer cell line HeLa was more resistant towards the tachyplesin peptides compared to the melanoma cell lines and higher peptide concentrations were necessary to reach 50% cell death. Differences in cytotoxic activity against HeLa, compared to the control cell line HaCaT, can be observed for all peptides except TI and cTI. The cyclic analogues cTI–cTIII were approximately 2x more potent against HeLa than the parent peptides TI–TIII (*p* < 0.05).

The peptides have a similar selectivity for the melanoma cell lines at lower peptide concentrations which would induce ≤10% hemolysis in RBCs. At concentrations inducing ≤50% hemolysis in RBCs, the cyclic analogues cTI–cTIII were more selective. Overall, the most promising therapeutic range was observed for TIII, cTI and cTIII (Table 5).

### 2.5. Mechanistic Studies

Peptides with an amphipathic arrangement of charged and hydrophobic residues are known to act against bacterial and cancer cells via selective membrane targeting, penetration and/or lysis. To characterize how peptide-membrane interactions affect biological activity, we undertook detailed peptide-membrane binding studies that compare parent and cyclic tachyplesin peptides.

#### 2.5.1. Peptide Binding to Model Membranes

The ability of TI to bind to model membranes has been previously shown and a preference for negatively-charged membranes was found [31,46]. We were interested in comparing the membrane-binding properties of the parent tachyplesins versus the cyclic analogues to determine whether differences in membrane binding could explain the relative biological activities (see Table 5). Given the similar potencies among the three tachyplesins, and among the three cyclic analogues, we compared the interaction of TI and cTI, as representative of parent and cyclic tachyplesin peptides, respectively, with model membranes using surface plasmon resonance (SPR). Phospholipids containing PC-headgroups are the most common in the outer leaflet of the mammalian plasma membrane [54,55]; thus, we prepared model membranes composed of the zwitterionic POPC (1-palmitoyl-2-oleoyl-sn-glycero-3-phosphocholine), which forms fluid bilayers at 25 °C and mimics the overall fluidity and neutral surface of healthy eukaryotic cells [56]. Phospholipids containing the negatively-charged PS-headgroups are normally restricted to the inner leaflet in eukaryotic cell membranes but are exposed at the cell surface of cancerous cells [20,22,23]. Therefore, we used model membranes with 20% of POPS (1-palmitoyl-2-oleoyl-sn-glycero-3-phosphoserine), POPC/POPS (4:1 molar ratio), to represent the negatively-charged surface of cancer cells.

**Figure 4:**
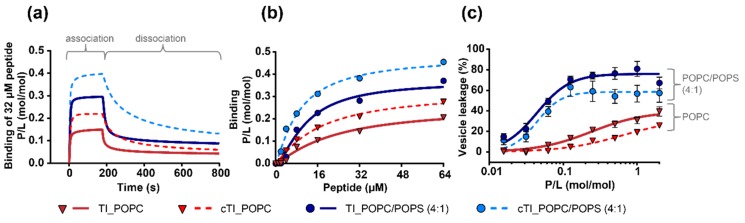
Membrane binding and disruption induced by tachyplesin I (TI) and cyclic tachyplesin I (cTI). Model membranes composed of 1-palmitoyl-2-oleoyl-sn-glycero-3-phosphocholine (POPC) and POPC/1-palmitoyl-2-oleoyl-sn-glycero-3-phosphoserine (POPS) (4:1) were compared. (**a**) Surface plasmon resonance sensorgrams obtained with 32 µM peptide injected over lipid bilayers deposited on an L1 chip surface for 180 s (association); dissociation was monitored for 600 s. Response units (RU) were converted into a peptide-to-lipid ratio (P/L (mol/mol)) to take into consideration the differences in lipid packing resulting in different amounts being deposited to cover the chip surface. (**b**) The dose-response curves show P/L obtained at the end of the association phase (*t* = 170 s) and plotted as a function of peptide concentration injected. (**c**) Percentage of vesicle leakage determined by fluorescence emission intensity of 5-carboxyfluorescein (*λ*_ex_ = 490 nm, *λ*_em_ = 513 nm) leaking from LUVs at increasing concentrations of peptide. Lipid concentration used was 5 µM, and the peptide was tested up to 10 µM. Dose-response curves were fitted with one-site specific binding with Hill slope equation in GraphPad Prism.

Both TI and cTI bound with higher affinity to negatively-charged POPC/POPS (4:1) than to zwitterionic POPC lipid bilayers. cTI had stronger affinity to both lipid systems than the parent peptide, as shown by a higher peptide-to-lipid (P/L) ratio during association, a slower dissociation rate (k_off_) from the lipids, and a higher amount of peptide remaining associated to the membrane at the end of dissociation (P/L_off_) (Figure 4a,b and Table 6). Additionally, TI and cTI disrupted large unilamellar vesicles (LUVs) of POPC/POPS (4:1) with higher efficacy than LUVs of POPC, which agrees with their preference for negatively-charged over neutral membranes. However, TI disrupted LUVs of POPC and of POPC/POPS (4:1) more efficiently than the cyclic analogue cTI (Figure 4c, Table 6). Thus, the higher binding saturation and affinity of cTI (see P/L_max_ and kinetic parameters in Table 6) to the membranes did not correlate with its ability to disrupt membranes. The higher efficacy of TI in disrupting membranes, compared to the cyclic analogue, might explain the higher activity of the parent TI–TIII for planktonic bacterial cells (see Table 3), compared to their cyclic analogues.

To examine the ability of cTI to bind model membranes that mimic bacterial cell membranes, we prepared vesicles with an *E. coli* polar lipid extract composed of zwitterionic phosphatidylethanolamine (PE)-phospholipids, negatively-charged phosphatidylglycerol (PG)-phospholipids, and cardiolipin (CA) in the proportion 67:23.2:9.8 (*wt*/*wt*%). The SPR sensorgram and dose-response curves (Supplementary Figure S3) show that cTI has a high affinity for *E. coli* lipids. Comparison of the dose-response curves and fitted parameters show that the maximum amount of cTI bound to *E. coli* lipids (P/L_max_, Supplementary Table S4) is not as high as for the other tested negatively-charged membranes or for the zwitterionic POPC, but the P/L_off_ is higher than from the other tested membranes (see Supplementary Table S4), suggesting that a large amount of peptide remains bound to the bilayers that mimic bacterial membranes. To investigate whether cTI distinguishes the negatively-charged headgroups present in bacteria (i.e., PG) from those in cancer cells (i.e., PS), we compared the binding of cTI to POPC/POPG (1-palmitoyl-2-oleoyl-sn-glycero-3-phosphoglycerol; 4:1)) and to POPC/POPS (4:1). cTI has a slightly higher affinity for model membranes containing PS-phospholipids than to those containing PG-phospholipids, as shown by the higher maximum amount of peptide bound to POPC/POPS (4:1), and a slower dissociation rate (see P/L_max_, k_off_ and P/L_off_ in Supplementary Figure S3).

#### 2.5.2. Partitioning of Trp Residue into Model Membranes

**Figure 5:**
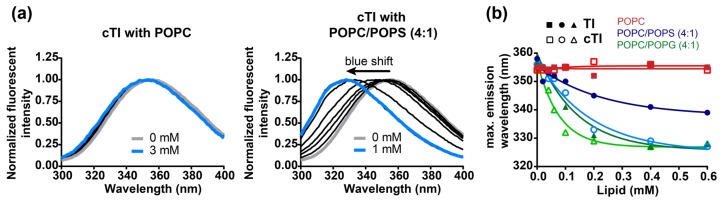
The partitioning of the 12.5 µM of tachyplesin I–III (TI-TIII) and cyclic analogues (cTI-cTIII) into lipid membranes. (**a**) Normalized fluorescence emission spectra of cTI in aqueous solution (grey) and upon titration with LUVs composed of POPC (1-palmitoyl-2-oleoyl-sn-glycero-3-phosphocholine; up to 3 mM) or of POPC/POPS (1-palmitoyl-2-oleoyl-sn-glycero-3-phosphoserine; 4:1) up to 1 mM). Fluorescence emission spectra were obtained with excitation at 280 nm (**b**) Wavelength at which TI (solid symbol) and cTI (open symbol) have their maximum fluorescence emission in buffer and with increasing concentrations of LUVs composed of different lipid mixtures (POPC: red; POPC/POPS (4:1): blue; POPC/POPG (1-palmitoyl-2-oleoyl-sn-glycero-3-phosphoglycerol; 4:1): green).

The fluorescence emission of Trp is sensitive to the local environment. In an aqueous environment, the fluorescence emission spectrum of Trp has a maximum at ~350 nm (*λ_ex_* = 280 nm), in a hydrophobic environment the fluorescence emission spectrum is blue-shifted and an increase in the fluorescence quantum yield is usually observed [57,58,59]. Tachyplesin peptides have one Trp residue: the fluorescence emission properties of this residue can be used to examine the environment surrounding it and inform on the partitioning and orientation of the peptides bound to model lipid bilayers. In the current study, we followed the changes in Trp fluorescence emission spectra of TI–TIII and of the cyclic analogues cTI–cTIII upon titration with LUVs composed of zwitterionic (POPC) or of negatively-charged membranes with two distinct negatively-charged headgroups, i.e., PS and PG (Figure 5).

TI and cTI bound to both zwitterionic POPC and negatively-charged POPC/POPS (4:1) bilayers, as shown with SPR (see Figure 4a,b); however, a blue shift in the Trp fluorescence emission spectra was only observed for model membranes containing negatively-charged phospholipids (Figure 5a, Table 7). Even at the highest concentration of 3 mM POPC LUVs, no change in the Trp fluorescence emission spectra was observed for any of the peptides. In contrast to POPC, a blue shift was detected when peptides were incubated with negatively-charged model membranes POPC/POPS (4:1) and POPC/POPG (4:1) at lipid concentrations as low as 0.1 mM (Figure 5).

In the presence of negatively-charged membranes, the Trp fluorescence emission maxima of cTI–cTIII had a larger shift than the respective parent peptides TI–TIII (Table 7), as illustrated with TI and cTI (Figure 5b). The differences between parent and cyclic peptides were most pronounced with POPC/POPS (4:1) bilayers: TI–TIII required more than twice the amount of POPC/POPS (4:1) LUVs compared to cTI–cTIII (0.19–0.30 mM vs. 0.08–0.12 mM) to induce half of the maximal shifts of the Trp fluorescence emission spectra (Figure 5b, Table 7). Comparison among parent peptides shows similar spectral shifts for a given model membrane (see for instance Trp fluorescence emission shifts of TI–TIII when titrated with POPC/POPS); the same is true among cyclic analogues. These results suggest that the mutations R/K1 or R/K15 have a weak influence, whereas backbone cyclization between R/K1 and G18 impacts the insertion of the Trp residue into lipid membranes.

The Trp fluorescence emission spectra of all tachyplesin analogues displayed larger blue shifts in the presence of POPC/POPG (4:1) compared to POPC/POPS (4:1) LUVs, which suggests that the Trp residue partitions better and/or inserts further into PG-containing membranes than into PS-containing membranes (Table 7). Despite the blue shift in the fluorescence emission spectra of all the peptides when in the presence of negatively-charged membranes, no increase in quantum yield was detected (data not shown). This could be explained by fluorescence quenching induced by neighboring amino acid side chains, local carbonyl groups [58], or through photon re-absorption between Trp residues of neighbor peptides molecules due to their closer proximity once inserted into lipidic membranes.

#### 2.5.3. Insertion of Trp Residue into Model Membranes

**Figure 6:**
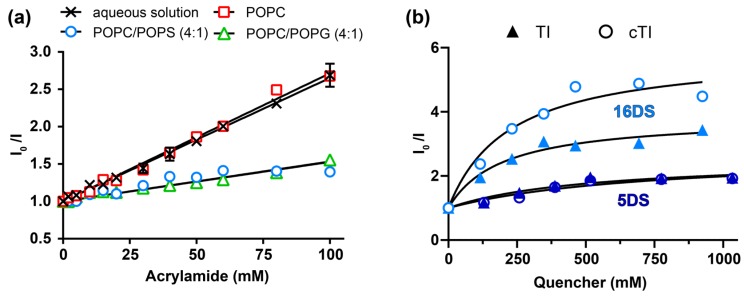
The effect of aqueous (acrylamide) and lipidic (5DS and 16DS) quenching on the tryptophan fluorescence intensity to investigate peptide in-depth location within model membranes. (**a**) The Trp fluorescence emission of 12.5 µM cTI in aqueous solution or in the presence of LUVs (1 mM POPC (1-palmitoyl-2-oleoyl-sn-glycero-3-phosphocholine), 1 mM POPC/POPS (1-palmitoyl-2-oleoyl-sn-glycero-3-phosphoserine; 4:1), 0.1 mM POPC/POPS (4:1) or 0.1 mM POPC/POPG (1-palmitoyl-2-oleoyl-sn-glycero-3-phosphoglycerol; 4:1)) in the absence (I_0_) and upon titration with increasing concentrations of acrylamide (I). Data are shown as Stern-Volmer plots (i.e., *I*_0_/*I* versus concentration of quencher). (**b**) Quenching of Trp fluorescence emission of 12.5 µM TI and cTI incorporated in 1 mM POPC/POPS (4:1) membranes by 5DS or 16DS.

The in-depth location of the Trp residue of tachyplesin peptides bound to lipid membranes was investigated using fluorescence quenching methodologies. Acrylamide, an aqueous quencher unable to partition into lipid bilayers, was used to quench the fluorescence of Trp residues exposed to the aqueous solution. If the Trp residue inserts into the lipid bilayer, it becomes inaccessible to quenching by the aqueous-soluble acrylamide. In addition to acrylamide quenching, we used the lipidic quenchers 5- and 16-doxyl stearic acids (5DS and 16DS) to gain information on the in-depth location of TI and cTI within the membrane. The acids 5- and 16DS are fatty acids that insert into the lipid bilayer and possess the quencher moiety, nitroxide, located at carbon 5 and 16 of the fatty acid chain, respectively. The proximity of the nitroxide moiety to the Trp residue is required for quenching of its fluorescence emission; thus, comparison between the 5- and 16DS quenching efficacies gives information on the location of the Trp residue when the peptide is partitioned into membranes [60,61]. Changes in the Trp fluorescence emission of individual peptides were followed upon titration with each of the quenchers. The fluorescence emission intensity in the absence and presence of the quencher (I_0_/I) were plotted as a function of the quencher concentration and used to determine the Stern-Volmer constants (K_SV_) (Figure 6), which are proportional to the accessibility of the quencher to the fluorophore.

A reduction in the slope (K_SV_) of the fitted data points in the presence of lipids, compared to the aqueous solution, indicates the reduced quenching efficacy by acrylamide. The percentage of Trp accessible to acrylamide was estimated by the ratio of K_SV_ obtained in the presence and absence of LUVs, assuming that the Trp residue is fully exposed to acrylamide when the peptide is in an aqueous solution. Acrylamide quenched the Trp fluorescence emission of both TI and cTI with a similar efficacy in the buffer and in the presence of 1 mM POPC LUVs (Figure 6a, Table 8). These results show that when TI and cTI are bound to POPC, their Trp residue is accessible to acrylamide and likely to be exposed to the aqueous environment. In contrast, when in the presence of 1 mM POPC/POPS (4:1), the Trp fluorescence emission of TI and cTI was not efficiently quenched by acrylamide, suggesting that their Trp residue is not accessible to acrylamide. A similar result is expected for all the analogues given the lack of insertion of the Trp residue of TI–TIII and of cTI–cTIII when bound to POPC membranes and the large blue shift in the fluorescence emission spectra of the Trp residue of all the analogues when bound to POPC/POPS (4:1) membranes (see Table 7).

To identify potential differences between the six analogues, we monitored the quenching of Trp fluorescence emission by acrylamide in the presence of 0.1 mM POPC/POPS (4:1) or of 0.1 mM POPC/POPG (4:1) membranes. In the presence of 0.1 mM POPC/POPS (4:1) LUVs, acrylamide quenched the Trp fluorescence emission of TI–TIII with a higher efficacy than that of the cyclic analogues, suggesting that the Trp residue of cTI–cTIII is more protected from acrylamide. The Trp of the native tachyplesin peptides was less accessible for quenching in the presence of 0.1 mM POPC/POPG (4:1) LUVs compared to 0.1 mM POPC/POPS (4:1) LUVs through insertion into the membrane. The accessibility of the acrylamide to the Trp residues of the cyclic analogues remained approximately the same with both lipid systems (Table 8). These results suggest that when compared at the same lipid concentrations, more TI–TIII are inserted into POPC/POPG (4:1), than into POPC/POPS (4:1) membranes, whereas the cyclic analogues did not distinguish between the two negatively-charged lipid mixtures.

**Figure 7:**
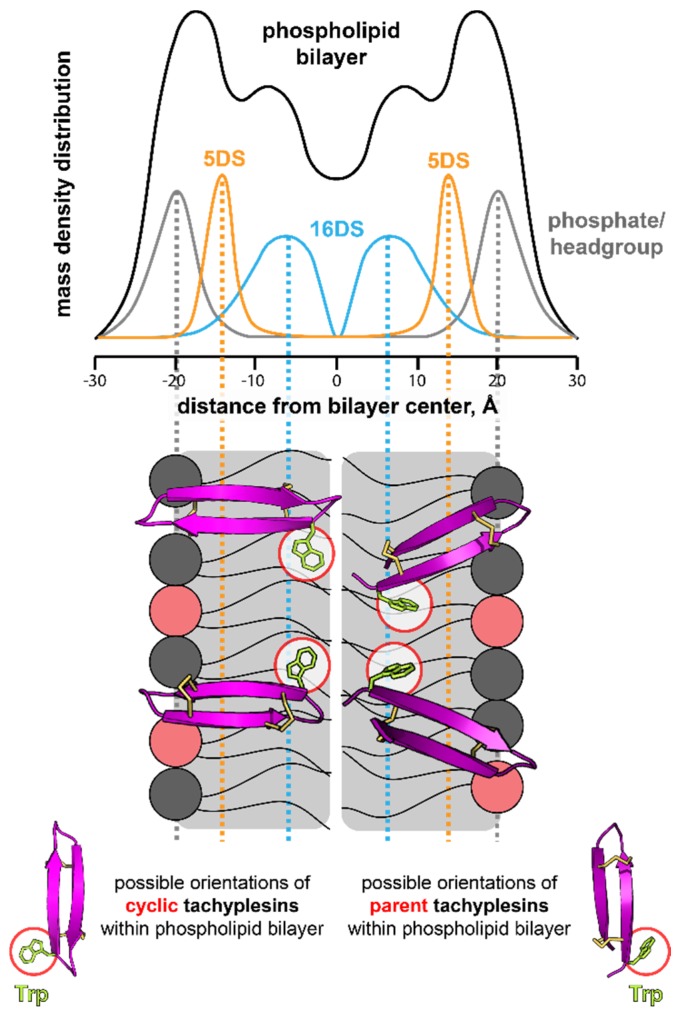
Schematic representation of the proposed orientations of tachyplesin peptides and cyclic analogues, when inserted into negatively-charged membranes based on the tryptophan fluorescence quenching, results with 5- and 16DS. The phospholipids in grey represent PC headgroups and the phospholipids in red represent PS headgroups. The peptides have a backbone length of approximately 25 Å (measured in PyMol) and are able to span across a lipid monolayer. Other orientations are possible. The distribution of the 5DS and 16DS quencher within a phospholipid bilayer was illustrated based on previous publications [60,63].

Comparison of the quenching efficacy by 5DS and 16DS tested with POPC/POPS (4:1) bilayers, showed that 16DS quenched the Trp fluorescence emission of TI and cTI with a higher efficacy than 5DS, which was particularly evident for the cyclic analogue (see Stern-Volmer plots and K_SV_ values in Figure 6b, Table 9). The higher quenching efficacy induced by 16DS suggests that the Trp residue of TI and of cTI is located within the hydrophobic core of the POPC/POPS (4:1) bilayers. The negative deviation to the linearity (see Figure 6b and f_b_ values in Table 9) suggests that the Trp residue of a fraction of peptide molecules is not accessible to the quenchers. This can be an indication of peptide molecules adopting different orientations within the membrane; nevertheless, the average location of the Trp residue of cTI and TI confirms that both peptides have their Trp residue deeply inserted and, in particular, the cyclic analogue. Overall, these results suggest that the Trp residue of cyclic analogues partitioned with a higher efficacy and/or were inserted deeper into anionic lipid bilayers, than parent tachyplesin peptides (Figure 7).

## 3. Discussion

In this study we demonstrated the high structural homology of the three tachyplesin peptides TI, TII and TIII. The primary sequence of these peptides differs only between Lys or Arg residues at positions 1 or 15, and both of these positions are located close to the flexible peptide termini. The cyclic analogues cTI, cTII and cTIII share a high structural homology with each other and with their respective parent peptide, but the backbone cyclization reduced the range of motion of the amino acid side chains located in the region of the termini (see Figure 1d).

TI–TIII and cTI–cTIII were active against representative Gram-positive and Gram-negative bacteria at low micromolar MICs (see Table 3). The antimicrobial activities of the three tachyplesin peptides were similar, as were the activities of the three cyclic analogues; however, differences were observed between the two groups. Overall, the parent tachyplesin peptides exhibited stronger antimicrobial activities than the cyclic analogues against all bacteria tested in the planktonic growth form. The Gram-negative *E. coli* strains were more sensitive to the peptides than the Gram-positive *S. aureus* strains. Against the *S. aureus* biofilm (see Figure 3 and Table 4), the tachyplesin peptides were >5-fold less active compared to the planktonic cells, likely due to the reduced accessibility of the peptides to the bacterial cell in biofilm form or the low ability of the peptides to penetrate the extracellular matrix of exopolysaccharides and access the cells [64]. The activities of the tachyplesin peptides against biofilms are comparable to other tested antimicrobial peptides [53]. The efficacy against the biofilms could be increased by using higher concentrations of the tachyplesin peptides or by using the peptide in combination with other antibiofilm or extracellular matrix disrupting agents [65]. Interestingly, TIII had been previously shown to prevent the formation of a *Pseudomonas aeruginosa* biofilm in a rat model of ureteral stent infection [66]; thus, even if the peptides cannot be used to completely disrupt the biofilm at the tested concentration, they could be useful for preventing biofilm formation on biomedical devices [67].

TI–TIII and cTI–cTIII peptides had a higher efficacy against the melanoma cell lines MM96L, HT144 and WM164 than to non-cancerous cells or the HeLa cervical cancer cell line (see Table 5). Interestingly, the cyclic tachyplesin analogues were more active against the cervical cancer cell line HeLa, than the parent tachyplesins. Differences in membrane composition between the different cells might explain the selectivity of the six analogues for melanoma over other cell lines [43], whereas a variation in the lipid-binding affinity and orientation within the membrane between parent versus cyclic analogues might explain more subtle differences observed against individual cell lines.

The experiments with model membranes confirmed the preference of all peptides for negatively-charged membranes (see Figure 4). TI, and its cyclized analogue cTI had a higher affinity for the negatively-charged POPC/POPS (4:1) than for neutral POPC bilayers, as shown in SPR experiments. cTI bond stronger and dissociated slower from POPC/POPS (4:1) and POPC bilayers than TI; however, TI disrupted both lipid bilayers with a higher efficacy than cTI. The ability of tachyplesin peptides to disrupt negatively-charged and neutral membranes with higher efficacy than their respective cyclic analogues, corroborates with their higher antimicrobial and hemolytic activities.

The fluorescence spectroscopy experiments show that the Trp residue of all six tachyplesin analogues partition and/or insert deeper into POPC/POPG (4:1), than into POPC/POPS (4:1) lipid bilayers (see Figure 5); in contrast, SPR studies show that cTI has a slightly higher affinity for POPC/POPS (4:1) than for POPC/POPG (4:1) membranes. This apparent contradiction emphasizes that specific phospholipid headgroups and membrane composition play a role in regulating and fine-tuning peptide-lipid binding interactions and the location of specific residues within the membrane. 

The orientation of the peptide bound to membranes might also be affected by the phospholipid headgroup. When TI–TIII and cTI–cTIII were bound to neutral model membranes, their tryptophan residue did not insert into the bilayer. These results support an orientation in which both parent and cyclic tachyplesin peptides are parallel to the water-lipid interface, or with an orientation in which the β-turn (Y8-I11) inserts into the hydrophobic region. In the presence of negatively-charged membranes, the Trp residue of both parent and cyclic tachyplesin peptides partitioned into the membrane. However, the Trp residue in cyclic peptides cTI–cTIII seemed to be more deeply inserted into the bilayer and less exposed to the aqueous environment (see Figure 6, Table 8 and Table 9).

Previous studies detected minor alterations to the peptide backbone of TI: the bending of the termini when the peptide was in contact with lipid membranes [36,68]. Doherty et al. [69] suggested that large amplitude motions of TI in the plane of the lipid membrane are essential for translocation, pore formation and membrane disruption. Since backbone cyclization impacts the flexibility of the termini region, cyclic tachyplesin analogues could be more restricted in the conformational changes of their backbone upon interaction with the membrane. The lower flexibility in the termini region might explain the lower ability of the cyclic analogue cTI to disrupt lipid bilayers compared to the parent tachyplesin. Furthermore, backbone cyclization created a second turn (Y17-W2) at which the Trp residue is located (see Figure 1d). The cyclic tachyplesin peptides are likely to insert deeply into the lipid membrane with the second turn (Y17-W2) inserting close to the center of the bilayer (see Figure 6 and Table 9), whereas in the parent peptides, the Trp residue is slightly closer to the membrane interface. A shallower location of the Trp residue could result from the parent peptides adopting a tilted orientation within the membrane (see Figure 7). Differences in the orientation of the peptides when inserted into lipid membranes could explain the higher membrane-disruptive properties and hemolytic activity of TI, compared to cTI (see Figure 4c and Table 6).

Backbone cyclization was shown to increase stability to proteolytic degradation, reduce membrane disruption and decrease hemolysis (see Figure 2). Interestingly, cTII has a slightly higher hemolytic activity compared to cTI and cTIII. cTII has an Arg residue at position 1 and a Lys at position 15, whereas cTI and cTIII have Lys residues at position 1. Arg and Lys residues establish different interactions with the phospholipid headgroups. The side chain of Arg residues can establish hydrogen bonds (H-bond) with two phospholipid headgroups, whereas the side chain in Lys residues can only form one H-bond. In contrast to Lys, Arg residues can form an H-bond through their side chains while being involved in cation-π-interactions [70]. Taking into consideration the increased toxicity of cTII due to an Arg residue, a Lys residue at position 1 favors the selectivity towards cancer cells.

In conclusion, TI–TIII and their cyclized analogues cTI–cTIII have high structural homology. Backbone cyclization increased peptide stability against proteolytic degradation, reduced the flexibility of amino acid side chains located in the terminal regions (i.e., R/K1, W2), and hemolytic activity while maintaining potent anticancer and antimicrobial activities. Since high resistance to proteolysis and low toxicity for host cells are preferred properties for peptide-based drug templates, the cyclic analogues have a higher potential than the parent counterparts, despite a possible loss of potency depending on the target. The Arg at position 1 of the amino acid sequence of TII and cTII proved to be non-beneficial for reducing hemolytic activity and improving selectivity for cancerous cells. Thus, cTI and cTIII have a higher potential than cTII as peptide-based anticancer drug templates. We also found that melanoma cell lines are more susceptible to the treatment with the tachyplesin peptides than the cervical cancer cell line HeLa. Systematic changes of the amino acid sequence might be applied to further increase the selectivity and/or anticancer activity of cyclic tachyplesin analogues (i.e. cTI or cTIII) to target specific cancers.

## 4. Material and Methods

### 4.1. Peptide Synthesis, Folding and Purification

The synthesis, folding and purification of synthetic peptides were carried out as previously described [42,71]. Briefly, the peptides were synthesized using 9-fluorenylmethoxycarbonyl (Fmoc) solid-phase peptide synthesis (SPPS) on an automatic peptide synthesizer (Symphony, Protein Technologies Inc., Tucson, USA). Rink amide resin was used for the synthesis of parent tachyplesin peptides and 2-chlorotrityl (2-CTC) resin for the backbone cyclized analogues. The peptides were oxidized overnight in 0.1 M ammonium bicarbonate buffer at pH 8.5 and purified using reverse-phase HPLC (solvent A: H_2_O, 0.05% (*v*/*v*) trifluoroacetic acid (TFA), solvent B: 90% (*v*/*v*) acetonitrile, 0.05% (*v*/*v*) TFA) until the desired purity of >95%. The correct peptide mass was confirmed with ESI-MS, while native disulfide connectivity was inferred from the dispersion of peaks in the 1D NMR spectra using a Bruker Avance 600 MHz spectrometer (Billerica, USA). The peptide concentration was determined from the absorbance at 280 nm (*ɛ_280_* = 8730 M^−1^.cm^−1^ as estimated extinction coefficient based on the contribution of Tyr and Trp residues, and disulfide bonds).

### 4.2. NMR Spectroscopy

For the structural analysis of TI–III and cTI–III, peptide (1 mg/mL) was dissolved in H_2_O/D_2_O (10:1, *v*/*v*) and the pH adjusted to pH 4–5. 1D ^1^H spectra, two-dimensional total correlated spectroscopy (TOCSY) and nuclear Overhauser effect spectroscopy (NOESY) were acquired with a Bruker Avance 600 MHz NMR spectrometer (Billerica, USA) at a temperature of 298 K. Additional spectra for ^1^H-^13^C HSQC and ^1^H-^15^N HSQC in H_2_O/D_2_O (10:1, *v*/*v*) and exclusive correlation spectra (E.COSY) in D_2_O were acquired. Spectra were referenced to an internal standard 2,2-dimethyl-2-silapentone-5-sulfonate (DSS) at 0 ppm. CYANA 3.97 was used to automatically calculate and refine structures based on distance restraints derived from the NOESY spectra [72], and torsion angles (ϕ and φ) generated using TALOS-N and Hα, Cα, Cβ, HN chemical shifts derived from NOESY, ^1^H-^13^C HSQC and ^1^H-^15^N HSQC spectra [73]. Several χ1 side-chain angle restraints were added based on E.COSY and NOESY data. A final set of structures was generated with CNS [74] using torsion angle dynamics, refinement and energy minimization in explicit solvent. Final structures were assessed for stereochemical quality using MolProbity [75].

### 4.3. Serum Stability

The serum stability assay was carried out as previously described [42] with some modifications. Briefly, tachyplesin variants were incubated in 25% (*v*/*v*) human serum diluted in phosphate-buffered saline (PBS) at a final concentration of 50 µM at 37 °C. Triplicates were collected at time 0 h and 24 h and the serum proteins were precipitated with acetonitrile (1:3 ratio) supplemented with 3% TFA. Samples were kept on ice for 10 min before centrifugation at 17,000× *g* for 10 min at 4 °C. The peptide containing supernatant of each sample was harvested and quantified using RP-HPLC (10 to 45% solvent B, 1%/min gradient). The percentage of peptide stability in human serum was calculated by comparing the area of the peptide peak obtained at 24 h to that at time 0 h. A linear peptide containing 18 amino acid residues (KGGGGSGQLIDSMANSFV) was included as a control susceptible to proteolytic degradation.

### 4.4. Hemolytic Studies

A small amount of blood was collected from three healthy human donors. The blood was immediately diluted in PBS and centrifuged 4–5 times for 1 min at 4000 rpm to wash and separate the human red blood cells (RBCs). RBCs suspension (0.25% (*v*/*v*) in PBS) was incubated with peptides with two-fold serial dilutions of the peptide (highest concentration tested was 128 µM, and the lowest was 0.25 µM) in a 96-well plate. Melittin, a membrane disruptive peptide, was used as control. The plates were incubated for 1 h at 37 °C. After incubation, the plates were centrifuged for 5 min at 1000 rpm to pellet any non-lysed RBCs. A total of 100 µL of the supernatant were transferred to a new 96-well plate [76]. The hemoglobin released into the supernatant from lysed cells was measured by absorbance at 415 nm using the Tecan infinite M1000Pro multiplate reader (Männedorf, Switzerland).

### 4.5. Cell Culture

Cells were grown in cell culture flasks and incubated in a humidified atmosphere (5% CO_2_, 37 °C). The cancer cell lines HeLa and the control cell line HaCaT were grown in a DMEM medium supplemented with 1% (*v*/*v*) penicillin/streptomycin and 10% (*v*/*v*) fetal bovine serum (FBS). The cancer cell lines MM96L, HT144 and WM164 were grown in a RPMI medium supplemented with 1% (*v*/*v*) penicillin/streptavidin, 10% (*v*/*v*) FBS, 20 mM l-glutamine, and 10 mM sodium pyruvate. Cell cultures were maintained by dilution upon reaching confluence, each 48–72 h. More information about the cell lines [77] is available in Tables S2 and S3.

### 4.6. Cytotoxicity Assays

Cells were seeded into 96-well flat-bottom plates at 5 × 10^3^ cells/well and incubated overnight. The medium was removed and replaced with 90 µL serum-free medium, 10 µL of 10x concentrated peptide solutions in PBS were added. PBS was added as blank and 0.1% (*v*/*v*) Triton X-100 was used to establish 100% of cell death. After 2 h incubation at 37 °C, 10 µL of filtered 0.05% (*w*/*v*) resazurin solution was added to each well [70]. Resazurin is converted to the pink and fluorescent compound resorufin by viable cells [78]. After incubation overnight, the fluorescence intensity (*λ*_ex_ = 565 nm and *λ*_em_ = 584 nm) was measured with the Tecan infinite M1000Pro multiplate reader (Männedorf, Switzerland).

The selectivity was calculated through the activity-toxicity index (ATI) [46]. ATI = MHC/MCC50, with MHC being the minimal concentration necessary to induce 10% or 50% cell death in human red blood cells and MCC50 the median of cytotoxic concentrations (CC50) of all tested melanoma cell lines.

### 4.7. Antimicrobial Studies

*S. aureus* ATCC 25923, *S. aureus* ATCC 6538, *E. coli* ATCC 25922, and *E. coli* DC2 CGSC 7139 were grown in Mueller Hinton Broth (Sigma Aldrich, St. Luis, USA). Bacterial cultures in the exponential growth phase were diluted to an OD600nm of 0.001 and seeded into 96-well plates. Peptides at different concentrations, starting at 64 µM and with two-fold serial dilutions (i.e., 64, 32, 16, 8, 4, 2, 1, 0.5, 0.25, 0.125 and 0.0625 µM), were incubated with cells [43]; 0.05% (*v*/*v*) resazurin was added to the cultures the next day and the conversion to resorufin was measured after 1 h of incubation at 37 °C using a 565 nm excitation and 584 nm emission wavelength, as above.

### 4.8. Biofilm Studies

*S. aureus* ATCC 6538 (1 × 10^6^ cfu/mL) was cultured in Tryptic Soy Broth, containing 0.25% (*w*/*v*) glucose and incubated in 96-well microtiter flat-bottomed polystyrene plates for 24 h at 37 °C. Preformed biofilms were then washed with Mueller Hinton Broth to remove non-adherent cells. Two-fold serial dilutions of each peptide (highest concentration tested was 32 µM and lowest was 0.25 µM) were added to the biofilms for 4 h. Untreated 24 h preformed biofilms were used as a control. The metabolic activity of biofilm-embedded cells was determined using a resazurin reduction fluorometric assay as previously described [53].

### 4.9. Lipid Vesicle Preparation

Mixtures with synthetic lipids (POPC, POPS, POPG, Avanti Polar Lipids) or *E. coli* lipid extract (Avanti Polar Lipids, Alabaster. USA) were extruded in HEPES buffer (10 mM HEPES, 150 mM NaCl, pH 7.4) to produce lipid vesicles, as previously described [43,79]. LUVs (Ø ≤ 100 nm) were used in fluorescence spectroscopy assays and small unilamellar vesicles (SUVs, Ø ≤ 50 nm) for SPR.

### 4.10. Fluorescence Spectroscopy Assays

Fluorescence emission spectra (300–400 nm, excitation at 280 nm, slits 3/3 mm) of 12.5 µM peptide and l-Trp in HEPES buffer (in quartz cuvettes, path length of 0.5 cm) were scanned upon titration with LUVs composed of various lipid compositions (up to 3 mM POPC, and up to 1.5 mM POPC/POPS (4:1) or POPC/POPG (4:1)) [80] using a FluoroMax-4 spectrofluorometer (Horiba, Kyoto, Japan). Integrated areas of the fluorescence emission spectra were corrected for fluorophore dilution and light dispersion due to titration with LUVs suspension; the blank was discounted.

### 4.11. Quenching of Tryptophan Fluorescence

The membrane in-depth location of the Trp residue within the peptides was followed using Acrylamide (Sigma Aldrich, St. Luis, USA) and 5- and 16-DS (Sigma Aldrich, St. Luis, USA). Quenching induced by acrylamide was monitored with 12.5 µM peptide in a HEPES buffer, in the presence of 1 mM of POPC, 0.1 mM POPC/POPS (4:1), 1 mM POPC/POPS (4:1), or 0.1 mM POPC/POPG (4:1) titrated with increasing concentration of acrylamide [81]. The fluorescence emission spectra were determined with an excitation wavelength of 290 nm to reduce the quencher/fluorophore light absorption ratio. The fluorescence emission spectra area was corrected for the inner filter effect [82] due to increased absorbance of acrylamide. Data points were analyzed using the Stern-Volmer representation (Equation (1)), the K_SV_ is determined from the slope.

(1) $$\frac{I_{0}}{I}=1+K_{SV}\left[Q\right]$$

A total of 12.5 µM of TI, or of cTI, in HEPES buffer and 1 mM POPC/POPS (4:1) were titrated with increasing concentration of 5DS, or of 16DS. The fluorescence emission spectra area was corrected for the inner filter effect [82] due to increased absorbance of 5-,16DS. The effective concentration of 5DS and 16DS in the lipid bilayer was calculated: partition coefficients of 5DS and 16DS into fluid membranes are 89,000 and 9730, respectively [59,81]. The data points had a negative deviation to linearity and the K_SV_ was determined by fitting the data with the Lehrer equation (Equation (2)) [59,81].

(2) $$\frac{I_{0}}{I}=\frac{1+K_{SV}\left[Q\right]}{\left(1+K_{SV}\left[Q\right]\right)\left(1-f_{B}\right)+f_{B}}$$

*I*_0_ = fluorescent intensity in buffer

I = fluorescent intensity in presence of quencher

*K*_SV_ = Stern-Volmer constant

[*Q*] = quencher concentration

*f*_B_ = fraction of light accessible to the quencher = $\frac{I_{0,B}}{I_{0}}$

*I*_0,B_ = fluorescent intensity of the accessible population of the quencher when [Q] = 0

The average distance of the Trp residue from the bilayer center (Å) was determined using the parallax method [62] following the equation,

(3) $$z_{1F}=\frac{\left(\frac{1}{-\pi C}\ln\frac{F_{1}}{F_{2}}-L_{21}^{2}\right)}{2L_{21}}$$

(4) $$z_{cF}=z_{1F}+L_{c1}$$

in which the *z*_1*F*_ is the distance between the Trp residue and the quencher moiety in 5DS, *L*_21_ is the distance between the quencher groups in 5 and 16DS (5 Å), F_1_ is the fluorescence of 12.5 µM of TI, or of cTI, in the presence of 1 mM POPC/POPS (4:1) and 0.4 mM 5DS, F_2_ is the fluorescence of 12.5 µM of TI, or of cTI, in the presence of 1 mM POPC/POPS (4:1) and 0.4 mM 16DS. C is the molar fraction of quencher within the total lipid concentration per unit area (assuming the surface of lipid to be 70 Å^2^) [62]. z_cF_ is the distance between the Trp residue and the center of the bilayer, L_*c*1_ is the distance between the center of the bilayers and the quencher group of 5DS (15 Å).

### 4.12. Surface Plasmon Resonance (SPR)

SPR was used to investigate the affinity and binding kinetics of peptides to membranes of different compositions. The experiments were conducted with an L1 biosensor chip (GE Healthcare) at 25 °C using a BIAcore 3000 instrument (GE Healthcare, Chicago, USA) [83,84]. The HEPES buffer was used for sample preparation and as a running buffer. Lipid bilayers were immobilized onto L1 chip by injection of SUVs at a flow rate of 2 µL/min. Peptide samples with two-fold serial dilutions (the highest concentration tested was 64 µM and the lowest was 1 µM) were injected over the lipid bilayer at a flow rate of 5 µL/min. Association of the peptides onto the lipid bilayer was followed for 180 s and the dissociation from the lipid for 600 s. The BIAeval software was used to analyze the sensorgrams. The response units (RU) were normalized to the peptide-to-lipid ratio (P/L); the P/L obtained at a fixed time point at the end of the association curve (at 170 s), at which the response has reached a plateau and the binding is close to equilibrium, was used to compare the affinity of the peptides to the different lipid systems [84].

### 4.13. Vesicle Leakage Assay

LUVs (Ø ≤ 100 nm) were prepared with the HEPES buffer containing 40 mM of the fluorescent 5-carboxyfluorescein (CF, Sigma Aldrich, St. Luis, USA). LUVs filled with CF at self-quenching concentrations were separated from the non-encapsulated dye on a Sephadex G-50 column equilibrated with HEPES buffer [85]. The concentration of CF-LUVs was determined through a calibration curve prepared from the original lipid mixture using Stewart’s assay (absorbance at 485 nm) [86]. Peptides were incubated (25 °C, 20 min) at two-fold dilutions (starting at 10 µM) with LUVs (5 µM) in the HEPES buffer in a 96-well flat-bottom black optiplates (Perkin Elmer, Waltham, USA). Vesicles and peptides were incubated for 20 min in dark and the release of CF was measured in a Tecan infinite M1000Pro multiplate reader using 490 nm as the excitation and 513 nm as emission wavelengths.

### 4.14. Statistical Analysis

Values (mean or fit ± SEM) were analyzed in GraphPad Prism 7 to test for significant differences in the cytotoxic activity of the peptides. The multiple t-test function with the Holm–Sidak method was applied when indicated. P values below 0.05 were considered to be significant.

## Figures and Tables

**Table 1:** The sequence and physicochemical properties of tachyplesin I–III (TI-TIII) and their cyclic analogues (cTI-cTIII).

Peptide	Sequence ^1^	Mass (Da) ^2^		RT (min) ^3^	Charge ^4^
		Calc.	Obs.		
**TI**	KWCFRVCYRGICYRRCR *	**2263.8**	2263.5	18.04	+7
TII	**R**WCFRVCYRGICYR**K**CR *	2263.8	2263.5	17.79	+7
TIII	KWCFRVCYRGICYR**K**CR *	2235.8	2235.6	17.68	+7
cTI	KWCFRVCYRGICYRRCR**G**	2303.8	2303.7	18.69	+6
cTII	**R**WCFRVCYRGICYR**K**CRG	2303.8	2303.7	18.53	+6
cTIII	KWCFRVCYRGICYR**K**CRG	2275.8	2275.5	18.44	+6

^1^ Peptide sequences and amino acid residues differing from TI are in bold. * denotes C-terminal amidation. ^2^ Average mass calculated (Calc.) from the amino acid sequence and experimentally observed (Obs.) using synthetic peptide and determined from m/z 3+ in ESI-MS. ^3^ Retention time (RT) of peptides on an analytical RP-HPLC; chromatograms shown in Supplementary Figure S1a were obtained with a 2%/min gradient of 0–40% solvent B (90% acetonitrile; 0.05% trifluoroacetic acid (TFA) (*v*/*v*)) in solvent A (H_2_O, 0.05% TFA (*v*/*v*)) at a flow rate of 0.3 mL/min. ^4^ Charge of the peptides at pH 7.4.

**Table 2:** Concentration of tachyplesin I–III (TI-TIII) and of their cyclic analogues (cTI-cTIII) required to induce 50% lysis in RBCs (HC50). ^a.^

Peptide	HC50 (µM)
TI	34.9 ± 2.8
TII	55.4 ± 6.6
TIII	86.4 ± 12.2
cTI	106.9 ± 21.0
cTII	64.1 ± 9.4
cTIII	>128

^a^ Values were determined from a minimum of three independent replicates and depicted as mean ± SEM.

**Table 3:** The minimal inhibitory concentrations (MICs) of tachyplesin I–III (TI-TIII) and of their cyclic analogues (cTI-cTIII) against planktonic bacteria.

	MIC (µM)			
Peptide	*E. coli* DC2 CGSC 7139	*E. coli* ATCC 25922	*S. aureus* ATCC 25923	*S. aureus* ATCC 6538
TI	0.5–1	0.0625–0.5	1–4	4–8
TII	0.5–1	0.125–0.5	1–4	4–8
TIII	0.25–0.5	0.0625–0.5	1–4	4–8
cTI	4	1	2–8	8
cTII	4–8	1–2	2–8	8
cTIII	4–8	1–2	2–8	8

**Table 4:** Activity of tachyplesin I–III (TI–TIII) and cyclic analogues (cTI–cTIII) against *S. aureus* ATCC 6538 in the biofilm growth form.

Peptide	CC50 (µM) ^1^	Metabolically Active Cells (%) ^2^
TI	21.5 ± 2.2	37.7 ± 4.3
TII	23.1 ± 2.9	39.9 ± 2.9
TIII	24.2 ± 4.1	43.3 ± 4.1
cTI	17.8 ± 3.6	31.9 ± 6.0
cTII	19.4 ± 1.5	34.6 ± 7.1
cTIII	18.0 ± 1.4	37.5 ± 7.2

^1^ Peptide concentration required to induce the reduction of 50% of the metabolically active cell population (CC50) determined using one-site specific binding with the Hill slope. ^2^ Percentage of cells remaining metabolically active at 32 µM, the highest peptide concentration tested.

**Table 5:** The cytotoxicity of tachyplesin I–III (TI-TIII) and cyclic analogues (cTI-cTIII) against cultured cells.

CC50 (µM) ^1^						Melanoma Selectivity ^5^
Peptide	MM96L ^2^	HT144 ^2^	WM164 ^2^	HeLa ^3^	HaCaT ^4^	
**TI**	1.5 ± 0.1	1.7 ± 0.2	2.5 ± 0.1	13.1 ± 1.2	11.6 ± 1.6	2–21
**TII**	1.6 ± 0.1	2.0 ± 0.1	1.6 ± 0.1	18.0 ± 3.9	3.7 ± 0.2	3–35
**TIII**	1.8 ± 0.1	2.0 ± 0.1	1.7 ± 0.1	21.7 ± 1.1	7.3 ± 0.5	5–48
**cTI**	1.3 ± 0.1	1.4 ± 0.1	2.7 ± 0.1	6.7 ± 0.6	7.9 ± 0.5	2–76
**cTII**	1.1 ± 0.1	0.8 ± 0.04	2.4 ± 0.3	7.2 ± 0.4	2.4 ± 0.3	3–58
**cTIII**	1.7 ± 0.1	0.9 ± 0.03	1.3 ± 0.1	9.3 ± 0.4	7.5 ± 0.3	3–98

^1^ The concentration necessary to kill 50% of cells was calculated from dose-response curves (n ≥ 3, ± SEM). ^2^ Melanoma cell lines: MM96L, HT144, WM164, ^3^ cervical cancer cell line: HeLa, ^4^ healthy epithelial control cell line: HaCaT (aneuploid immortal keratinocyte). Description and verification of each cell line are detailed in Supplementary Tables S2 and S3). ^5^ selectivity for melanoma cell lines was estimated through the activity-toxicity index (ATI). ATI = MHC/MCC50 (modified from Reference [46]), with MHC being the minimal concentration necessary to induce 10% (lower value) or 50% (higher value) cell death in human RBCs and MCC50 being the median of CC50 values of all melanoma cell lines. Values above 1 indicate a higher selectivity for the cancerous cells over RBCs.

**Table 6:** The kinetic and affinity parameters from surface plasmon resonance analysis of the interaction of 32 µM tachyplesin I (TI) and cyclic tachyplesin I (cTI) with neutral (POPC) and negatively-charged model membranes (POPC/POPS (4:1)) and leakage induced by the same peptides and lipid systems.

Peptide	Lipid System	P/L_max_ (mol/mol) ^1^	K_D_ (µM) ^1^	k_off_ (x 10^−2^ s^−1^) ^2^	P/L_off_ (mol/mol) ^2^	LC_max_ (%) ^3^
TI	POPC	0.26 ± 0.06	22.4 ± 10.9	1.50 ± 0.11	0.046 ± 0.001	39.5 ± 4.6
cTI		0.33 ± 0.07	16.7 ± 8.4	0.91 ± 0.03	0.065 ± 0.001	32.9 ± 9.4
TI	POPC/POPS (4:1)	0.37 ± 0.04	11.8 ± 2.4	2.75 ± 0.22	0.096 ± 0.001	76.0 ± 2.8
cTI		0.48 ± 0.08	9.2 ± 3.4	0.70 ± 0.03	0.137 ± 0.002	58.5 ± 3.6

^1^ P/L_max_ and K_D_ were calculated from the dose-response curves (one-site specific binding with Hill slope equation, GraphPad Prism) in Figure 4b. The P/L_max_ value represents the peptide-to-lipid ratio (mol/mol) when peptide-lipid binding reaches saturation, K_D_ is the peptide concentration necessary to reach the half-maximal binding response. ^2^ k_off_ is the dissociation constant and P/L_off_ is the peptide-lipid ratio at the end of association phase calculated from the sensorgrams obtained with 32 µM peptide in Figure 4a. k_off_ and P/L_off_ were fitted in GraphPad Prism, assuming a Langmuir kinetic. ^3^ Percentage of leakage achieved when incubating 10 µM peptide with 5 µM LUVs. POPC is 1-palmitoyl-2-oleoyl-sn-glycero-3-phosphocholine; POPS is 1-palmitoyl-2-oleoyl-sn-glycero-3-phosphoserine.

**Table 7:** Shift in the fluorescence emission maximum wavelength of tachyplesin I–III (TI-TIII) and cyclic analogues (cTI-cTIII) in buffer and in the presence of model membranes.

	POPC	POPC/POPS (4:1)		POPC/POPG (4:1)	
Peptide	shift (nm) ^1^	shift (nm)	0.5 [L] (mM) ^2^	shift (nm)	0.5 [L] (mM)
TI	2	22	0.19	30	0.09
TII	0	19	0.30	29	0.09
TIII	−3	18	0.30	23	0.10
cTI	0	28	0.11	28	0.06
cTII	1	27	0.08	29	0.05
cTIII	3	27	0.12	28	0.08

^1^ blue shifts observed with 3 mM POPC (1-palmitoyl-2-oleoyl-sn-glycero-3-phosphocholine); 1 mM POPC/POPS (1-palmitoyl-2-oleoyl-sn-glycero-3-phosphoserine; 4:1), or 1 mM POPC/POPG (1-palmitoyl-2-oleoyl-sn-glycero-3-phosphoglycerol; 4:1) LUVs. ^2^ lipid concentrations required to achieve half of the maximum blue shift observed (0.5 [L] in mM).

**Table 8:** Fluorescence quenching of 12.5 µM tachyplesin I-III (TI-TIII) or cylic tachyplesin I-III (cTI-cTIII) by acrylamide.

	Acrylamide Accessibility (%) ^1^			
	1 mM	1 mM	0.1 mM	0.1 mM
Peptide	POPC	POPC/POPS (4:1)	POPC/POPS (4:1)	POPC/POPG (4:1)
TI	90.5 ± 4.3	16.2 ± 21.9	72.3 ± 3.9	54.2 ± 5.8
TII			76.2 ± 3.2	56.8 ± 6.9
TIII			82.1 ± 4.7	75.4 ± 4.6
cTI	103.8 ± 2.3	22.5 ± 14.3	32.3 ± 8.3	32.0 ± 4.1
cTII			61.4 ± 5.5	36.1 ± 7.7
cTIII			48.7 ± 6.5	46.2 ± 5.7

^1^ Tryptophan accessibility of tachyplesin I-III (TI–TIII) and cyclic tachyplesin (cTI–cTIII) in the presence of LUVs (1 mM POPC (1-palmitoyl-2-oleoyl-sn-glycero-3-phosphocholine), 1 mM POPC/POPS (1-palmitoyl-2-oleoyl-sn-glycero-3-phosphoserine; 4:1), 0.1 mM POPC/POPS (4:1) or 0.1 mM POPC/POPG (1-palmitoyl-2-oleoyl-sn-glycero-3-phosphoglycerol; 4:1)) to the aqueous quencher acrylamide. The accessibility of the Trp residue to the quencher was calculated from the Stern-Volmer constants, K_SV_, which were derived from the linear fit of the data points (Equation (1)) of the peptide in buffer and peptide in the presence of lipids (see Figure 6a). Full exposition (100%) of the Trp residue to an aqueous environment was assumed in the buffer.

**Table 9:** Fluorescence quenching of tachyplesin I (TI) and and cyclic tachyplesin I (cTI) partitioned into 1 mM POPC/POPS (4:1) LUVs by the quenchers 5DS and 16DS. ^1.^

	5DS		16DS		
Peptide	K_SV_ (M^−1^)	f_b_	K_SV_ (M^−1^)	f_b_	Z (Å) ^2^
TI	3.7 ± 1.3	0.63 ± 0.57	19.6 ± 4.3	0.74 ± 0.37	8.8
cTI	4.9 ± 1.8	0.61 ± 0.54	26.3 ± 7.1	0.83 ± 0.52	5.5

^1^ The K_SV_ and f_b_ (the fraction of light emitted by the peptide accessible to the quencher) were determined using the Lehrer equation (Equation (2)). ^2^ The calculated average distance, Z in Å, of the Trp residue from the bilayer center was determined using the parallax method (Equations (3) and (4)) [62].

## Supplementary Materials

Supplementary materials can be found at https://www.mdpi.com/1422-0067/20/17/4184/s1.

## Abbreviations

1D	One-dimensional
2-CTC	2-chlorotrityl resin
3D	Three-dimensional
ACN	Acetonitrile
ATI	Activity/toxicity index
ATTC	American Type Culture Collection
BMRB	Biological magnetic resonance data bank
CC	Cytotoxic concentration
CF	5-carboxyfluorescein
cTI	Cyclic Tachyplesin I
cTII	Cyclic Tachyplesin II
cTIII	Cyclic Tachyplesin III
cTI–cTIII	Cyclic Tachyplesin I, II and III
DS	Doxyl-stearic acid
E.COSY	Exclusive correlation spectra
ESI-MS	Electrospray ionization mass spectroscopy
FBS	Fetal bovine serum
Fmoc	9-fluorenylmethoxycarbonyl
HC50	Hemolytic concentration necessary for 50% lysis of RBCs
HDP	Host defense peptide
K_SV_	Stern – Volmer constant
LPS	Lipopolysaccharide
LUV	Large unilamellar vesicle
MCC	Minimal cytotoxic concentration
MHC	Minimal hemolytic concentration
MIC	Minimal inhibitory concentration
NMR	Nuclear magnetic resonance
NOESY	Nuclear Overhauser effect spectroscopy
PBS	Phosphate buffered saline
PDB	Protein data bank
PC	Phosphatidylcholine
PS	Phosphatidylserine
PG	Phosphatidylglycerol
P/L	Peptide-to-lipid ratio
POPC	1-palmitoyl-2-oleoyl-glycero-3-phosphocholine
POPS	1-palmitoyl-2-oleoyl-sn-glycero-3-phospho-L-serine
POPG	1-palmitoyl-2-oleoyl-sn-glycero-3-phospho-(1′-rac-glycerol)
RBCs	Red blood cells
RP-HPLC	Reverse-phase high-performance liquid chromatography
RT	Retention time
RU	Response unit
SEM	Standard error of mean
SPPS	Solid phase peptide synthesis
SPR	Surface plasmon resonance
SUV	Small unilamellar vesicle
TI	Tachyplesin I
TII	Tachyplesin II
TIII	Tachyplesin III
TI–TIII	Tachyplesin I, II and III
TFA	Trifluoroacetic acid
TOCSY	Total correlated spectroscopy
