# Supplementary material for: Characterization of Tachyplesin Peptides and Their Cyclized Analogues to Improve Antimicrobial and Anticancer Properties
Source: Int J Mol Sci. 2019 Aug 26;20(17):4184. doi: 10.3390/ijms20174184 (PMC6747087; doi:10.3390/ijms20174184)
Supplement: Supplementary file 1 [file ijms-20-04184-s001.pdf]

Supplementary information

Characterization of tachyplesin peptides and their cyclized analogues to improve antimicrobial and anticancer properties

Felicitas Vernen[1], Peta J. Harvey[1], Susana A. Dias[2], Ana Salomé Veiga[2], Yen-Hua Huang[1], David J. Craik[1], Nicole Lawrence[1] and Sónia Troeira Henriques[1,3,*]

[1] Institute for Molecular Bioscience, The University of Queensland, QLD, 4072, Australia
[2] Instituto de Medicina Molecular João Lobo Antunes, Faculdade de Medicina, Universidade de Lisboa, 1649-028 Lisboa, Portugal
[3] School of Biomedical Sciences, Faculty of Health, Institute of Health & Biomedical Innovation, Queensland University of Technology, Translational Research Institute, Brisbane, Australia; sonia.henriques@qut.edu.au
* Correspondence: sonia.henriques@qut.edu.au; Tel.: +61 7 34437342

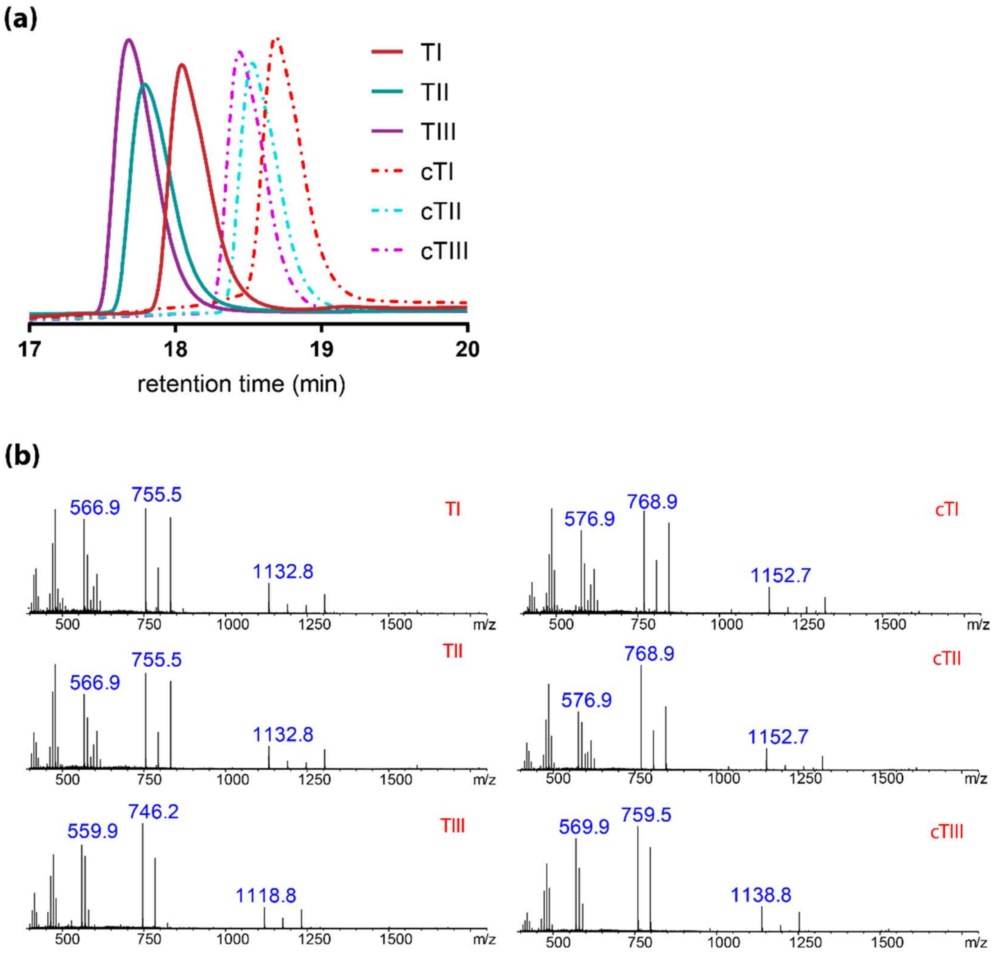

Figure S1. Purity and mass of tachyplesin analogues. (a) Chromatogram of the parent peptides (TI, TII, TIII) and their cyclic analogues (cTI, cTII, cTIII) on an analytical RP-HPLC obtained with a 2%/min gradient of 0–40% solvent B (90% acetonitrile (ACN); 0.05% trifluoroacetic acid (TFA) (v/v)) in solvent A (H_2O, 0.05% TFA (v/v)) at a flow rate of 0.3 ml/min. (b) Mass spectra of TI-TIII and cTI-cTIII determined by ESI-MS.

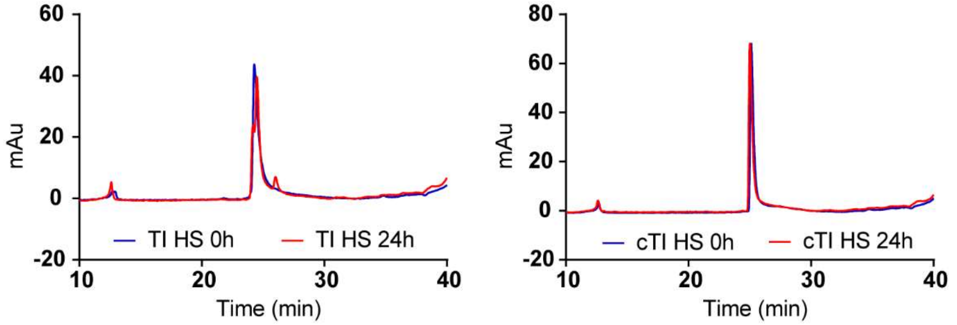

Figure S2. Serum stability of TI an cTI. Overlay of chromatograms obtained with 50 μM TI, or cTI, before (0h) and after incubation in 25% (v/v) human serum (HS) for 24 h with analytical RP-HPLC (10 to 45% solvent B, 1%/min gradient)

Table S1. Statistical analysis of linear and cyclic tachyplesin analogue structures. [a]

**Experimental restraints**	**tachyplesin II**	**tachyplesin III**	**cyclic tachyplesin I**	**cyclic tachyplesin II**	**cyclic tachyplesin III**
total no. distance restraints	154	154	284	172	230
intraresidue	56	51	124	53	72
sequential	38	37	87	41	79
medium range, *i-j*<5	34	33	42	47	55
long range, *i-j*≥5	26	33	31	31	24
dihedral angle restraints					
phi	16	15	15	16	16
psi	16	15	15	16	16
chi1	3	4	4	3	4
Deviations from idealized geometry					
bond lengths (Å)	0.011 ± 0.001	0.011 ± 0.000	0.011 ± 0.001	0.011 ± 0.001	0.011 ± 0.001
bond angles (deg)	1.128 ± 0.042	1.082 ± 0.044	1.139 ± 0.037	1.103 ± 0.041	1.053 ± 0.034
impropers (deg)	0.95 ± 0.11	0.99 ± 0.09	0.97 ± 0.09	1.01 ± 0.12	0.96 ± 0.10
NOE (Å)	0.009 ± 0.001	0.008 ± 0.002	0.007 ± 0.002	0.008 ± 0.003	0.007 ± 0.001
cDih (deg)	0.200 ± 0.093	0.256 ± 0.066	0.173 ± 0.086	0.139 ± 0.075	0.196 ± 0.086
Mean energies (kcal/mol)					
overall	-408 ± 13	-419 ± 10	-461 ± 8	-443 ± 12	-478 ± 7
bonds	9.1 ± 0.8	9.3 ± 0.8	9.3 ± 1.0	8.8 ± 1.0	9.5 ± 0.9
angles	19.9 ± 1.7	19.2 ± 1.7	20.9 ± 1.6	19.0 ± 1.6	18.4 ± 1.5
improper	6.5 ± 1.2	6.5 ± 0.8	6.8 ± 1.0	7.4 ± 1.3	6.8 ± 1.2
van Der Waals	-77.9 ± 4.7	-78.9 ± 3.8	-86.0 ± 3.0	-80.2 ± 4.1	-81.6 ± 3.5
NOE	0.01 ± 0.00	0.01 ± 0.00	0.02 ± 0.01	0.01 ±0.01	0.01 ±0.01
cDih	0.20 ±0.15	0.29 ± 0.15	0.15 ± 0.14	0.11 ± 0.11	0.20 ± 0.15
electrostatic	-444 ± 12	-452 ± 11	-495 ± 10	-481 ± 13	-514 ± 8
Violations					
NOE violations exceeding 0.2 Å	0	0	0	0	0
Dihedral violations exceeding 2.0 Å	0	0	0	0	0

Rms deviation from mean structure, Å					
backbone atoms	0.75 ± 0.21	0.73 ± 0.24	0.47 ± 0.15	0.52 ± 0.16	0.43 ± 0.17
all heavy atoms	2.17 ± 0.41	2.23 ± 0.39	1.33 ± 0.26	1.71 ± 0.31	1.47 ± 0.25
Stereochemical quality[b]					
Residues in most favoured Ramachandran region, %	100.0 ± 0.0	100 ± 0.0	99.7 ± 1.4	100.0 ± 0.0	100.0 ± 0.0
Ramachandran outliers, %	0 ± 0	0 ± 0	0 ± 0	0 ± 0	0 ± 0
Unfavourable sidechain rotamers, %	0 ± 0	0 ± 0	0 ± 0	0 ± 0	0 ± 0
Clashscore, all atoms	4.8 ± 1.9	5.8 ± 2.2	9.0 ± 2.7	6.2 ± 2.1	5.7 ± 2.8
Overall MolProbity score	1.2 ± 0.1	1.3 ± 0.1	1.5 ± 0.1	1.3 ± 0.1	1.3 ± 0.3

[a]All statistics are given as mean ± SD. [b]According to MolProbity [1]

Table S2. Origin of cell lines used in the study.

Cell line	Cancer type	Description	Cellosaurus ID [a]
MM96L	melanoma	Human, female, derived from metastatic site (lymph node), BRAF V600E mutant	CVCL_D853
HT144	melanoma	Human, male, 29 yr, Caucasian, derived from metastatic site (subcutaneous tissue), BRAF V600E mutant	CVCL_0318
WM164	melanoma	Human, male, 22 yr, Caucasian, derived from metastatic site, BRAF V600E mutant	CVCL_7928
HeLa	cervical cancer	Human, female, 31 yr, papillomavirus-related endocervical adenocarcinoma	CVCL_0030
HaCaT	keratinocyte	Human, male, 62 yr, spontaneously immortalized cell line	CVCL_0038

[a] Cellosaurus is a knowledge resource for cell lines used in biomedical research (https://web.expasy.org/cellosaurus/).

Table S3. Verification of cell line identity from Short Tandem Repeat (STR) profiles. [a]

Marker	Short Tandem Repeat Allele/s				
	MM96L	HT144	WM164	HeLa	HaCaT
D5S818	11,13	11,13	12	11,12	12
D13S317	11,14	11,12	11	12,13.3	10,12
D7S820	8	11	9,10	8,12	9,11
D16S539	11,12	12,13	12,13	9,10	9,12
vWA	17,18	16,18	14,15	16,17,18	16,17
TH01	7	9	9	7	9.3
Amel	X	X,Y	X	X	X
TPOX	8,10	8,11	8,10	8,12	11,12
CSF1PO	12	12	12	9,10	9,11

[a] STR profiles of the cell lines were verified against previously described database entries as follows: MM96L, QIMR, Brisbane Australia; WM164, Wistar; HT144, DSMZ-German collection of microorganisms and cell cultures GmBH; HeLa, ATCC; HaCaT, [2].

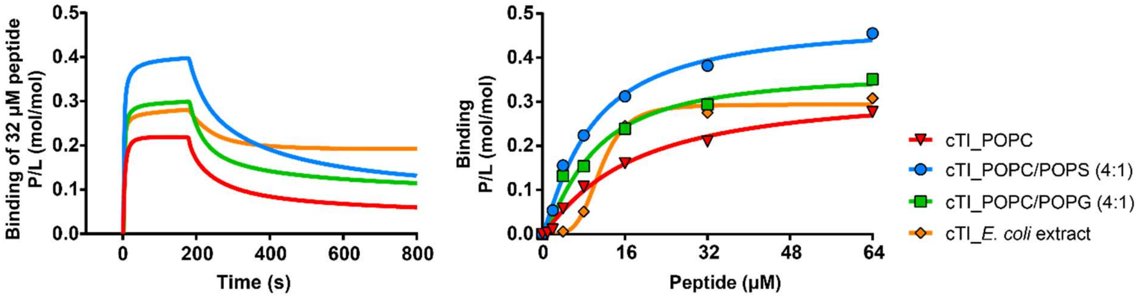

Figure S3. Binding of cTI to model membranes composed of POPC, POPC/POPS (4:1), POPC/POPG (4:1) and *E. coli* extract. Left panel: SPR sensorgrams obtained with 32 μM cTI injected over lipid bilayers deposited on an L1 chip surface for 180 s (association); dissociation was monitored for 600 s. Response units (RU) were converted into peptide-to-lipid ratio (P/L (mol/mol)) to take in consideration differences in lipid packing resulting in different amounts being deposited to cover the chip surface. Right panel: the dose-response curves show P/L obtained at the end of the association phase (t=170 s) and plotted as a function of peptide concentration injected. Dose-response curves were fitted with one-site specific binding with Hill slope equation in GraphPad Prism.

Table S4. Kinetic and affinity parameters from SPR analysis of 32 μM cTI with POPC, POPC/POPS (4:1), POPC/POPG (4:1) and *E. coli* extract [a].

Peptide	Lipid system	P/L_{max} (mol/mol)[1]	K_D (μM)[1]	k_{off} (x 10^2 s^{-1})[2]	P/L_{off} (mol/mol)[2]
cTI	POPC	0.33 ± 0.07	16.7 ± 8.4	0.91 ± 0.03	0.065 ± 0.001
	POPC/POPS (4:1)	0.48 ± 0.08	9.2 ± 3.4	0.70 ± 0.03	0.137 ± 0.002
	POPC/POPG (4:1)	0.37 ± 0.05	9.6 ± 3.1	1.09 ± 0.02	0.122 ± 0.001
	E. coli extract	0.29 ± 0.01	11.3 ± 0.5	1.31 ± 0.01	0.193 ± 0.001

[a] *E.coli* polar lipid extract composed of zwitterionic phosphatidylethanolamine (PE)-phospholipids, negatively-charged phosphatidylglycerol (PG)-phospholipids, and cardiolipin (CA) in the proportion 67:23.2:9.8 (wt/wt%)
[1]P/L_{max} and K_D were calculated from the dose-response curves (one-site specific binding with Hill slope equation, GraphPad Prism) in Figure S3. The P/L_{max} value represents the peptide-to-lipid ratio (mol/mol) when peptide-lipid binding reaches saturation, K_D is the peptide concentration necessary to reach the half-maximal binding response.
[2] k_{off} is dissociation constant and P/L_{off} is the peptide-to-lipid ratio that remains associated to the membrane at the end of association phase calculated from the sensorgrams obtained with 32 μM peptide in Figure S3 fitted in GraphPad Prism assuming a Langmuir kinetic.

References

1. Chen, V.B.; Arendall, W.B., 3rd; Headd, J.J.; Keedy, D.A.; Immormino, R.M.; Kapral, G.J.; Murray, L.W.; Richardson, J.S.; Richardson, D.C. MolProbity: all-atom structure validation for macromolecular crystallography. *Acta Crystallogr. D Biol. Crystallogr.* **2010**, *66*, 12-21, doi:10.1107/S0907444909042073.
2. Huang, Y.; Liu, Y.; Zheng, C.; Shen, C. Investigation of Cross-Contamination and Misidentification of 278 Widely Used Tumor Cell Lines. *PLoS One* **2017**, *12*, e0170384, doi:10.1371/journal.pone.0170384.
